# Self-prioritization in working memory gating

**DOI:** 10.3758/s13414-024-02869-8

**Published:** 2024-03-15

**Authors:** Roel van Dooren, Bryant J. Jongkees, Roberta Sellaro

**Affiliations:** 1 Cognitive Psychology Unit, Institute of Psychology and Leiden Institute for Brain and Cognition, Leiden University, Leiden, The Netherlands; 2 Department of Developmental Psychology and Socialization and Padova Neuroscience Center, University of Padova, Padova, Italy

**Keywords:** Working memory, Updating, Gating, Cognitive control, Self-prioritization, Self-bias

## Abstract

Working memory (WM) involves a dynamic interplay between temporary maintenance and updating of goal-relevant information. The balance between maintenance and updating is regulated by an input-gating mechanism that determines which information should enter WM (gate opening) and which should be kept out (gate closing). We investigated whether updating and gate opening/closing are differentially sensitive to the kind of information to be encoded and maintained in WM. Specifically, since the social salience of a stimulus is known to affect cognitive performance, we investigated if self-relevant information differentially impacts maintenance, updating, or gate opening/closing. Participants first learned to associate two neutral shapes with two social labels (i.e., “you” vs. “stranger”), respectively. Subsequently they performed the reference-back paradigm, a well-established WM task that disentangles WM updating, gate opening, and gate closing. Crucially, the shapes previously associated with the self or a stranger served as target stimuli in the reference-back task. We replicated the typical finding of a repetition benefit when consecutive trials require opening the gate to WM. In Study 1 (N = 45) this advantage disappeared when self-associated stimuli were recently gated into WM and immediately needed to be replaced by stranger-associated stimuli. However, this was not replicated in a larger sample (Study 2; N = 90), where a repetition benefit always occurred on consecutive gate-opening trials. Overall, our results do not provide evidence that the self-relevance of stimuli modulates component processes of WM. We discuss possible reasons for this null finding, including the importance of continuous reinstatement and task-relevance of the shape-label associations.

## Introduction

Working memory (WM) refers to the cognitive system that enables us to select and temporarily maintain goal-relevant information (i.e., maintenance) and to remove and replace this information as goals and/or circumstances change (i.e., updating) (Cowan, 2017; Hazy et al., 2006; Oberauer et al., 2018). Particularly influential neurocomputational WM models (Chatham & Badre, 2015; Frank et al., 2001; Hazy et al., 2006; O’Reilly & Frank, 2006; O’Reilly, 2006) have hypothesized the existence of an input-gating mechanism that regulates the balance between updating and maintenance by “deciding” which information is relevant and should enter WM, and which is not and should therefore be kept out of WM (Frank et al., 2001; O’Reilly & Frank, 2006). While a “gate open” state permits new input to gain access to WM and, when necessary, to replace outdated information, thereby enabling the updating of WM content, a “gate closed” state allows holding goal-relevant information in WM and shielding such content from irrelevant input. According to the prefrontal cortex basal ganglia working memory (PBWM) model (Frank & O’Reilly, 2006), input gating is implemented via dopamine (DA) signaling in basal ganglia (BG)-thalamus-prefrontal cortex (PFC) circuits, in keeping with mounting evidence that flexible goal-directed cognition depends on dopaminergic prefrontal-striatal projections (Cools and D’Esposito, 2011; Cools, 2019). Specifically, gate opening is thought to be prompted by phasic DA release in BG, which causes a disinhibition of the thalamus and a resulting activation of the PFC (Hazy et al., 2006). This causes information to flow into WM and updating to take place. Gate closing on the other hand is accomplished by a tonic inhibition of the thalamus and a resulting inhibition of the PFC, which facilitates the maintenance of information and prevents WM updating (Hazy et al., 2006).

Smooth and context-appropriate switching between these two functionally opposite states of WM (updating and maintenance) is necessary to accommodate the well-known WM capacity limitations (Miller, 1956) and enables successful interaction with a dynamically changing environment, in which distractions are numerous and relevant information continuously changes (Baddeley & Hitch, 1974; Baddeley, 1992; Cowan, 1999; Oberauer, 2009). Given the importance of WM to adaptive goal-directed behavior, accumulating empirical attempts have been made to gain a better understanding of the dynamics of WM by teasing apart its component processes (for an overview, see Trutti et al., 2021). The reference-back paradigm (Rac-Lubashevsky & Kessler, 2016a, 2016b) has recently been established as a well-suited task to study and isolate WM subprocesses in terms of gate opening versus closing, and updating versus maintenance of WM content.

The task (see *Methods* for more details) is an adapted version of the popular N-back paradigm and involves the presentation of a sequence of two or more distinct stimuli (e.g., a cat and an apple). On each trial, only one of the stimuli is presented, along with a task cue (e.g., a frame varying in terms of shape: circle vs. square) that instructs participants to maintain or update their WM content. Participants must always indicate whether the currently shown stimulus is the same as or different from the stimulus shown on the most recent updating trial (e.g., the most recent stimulus shown within a square frame). As such, each trial involves a matching judgment, but some trials additionally require WM updating (e.g., trials with a square frame, which show a stimulus that must be remembered for subsequent trials), whereas other trials require WM maintenance (e.g., trials with a circular frame, which show a stimulus that must not be remembered for subsequent trials). The reference-back literature typically refers to updating and maintenance trials as “reference” and “comparison” trials, respectively, to denote that the former requires encoding a new stimulus into WM whereas the latter only requires a comparison to be made between a currently shown stimulus and the one maintained in WM. As both reference and comparison trials involve matching decisions, but only the former demands WM updating, the performance decrement on reference trials relative to comparison trials is suggested to reflect the behavioral cost of *updating* WM content. Note that updating costs occur regardless of whether WM content has to be updated with a new item or an item that is already maintained in WM (Rac-Lubashevsky & Kessler, 2016b). However, updating WM content with new (as compared to already-maintained) information requires additional time (Oberauer et al., 2013), namely, the time that it takes to remove the information currently held in WM (here, the current referent) and to replace it with newly encoded information (here, the new referent).

The efficiency of gate-opening and gate-closing processes can be inferred by taking into consideration transitions between comparison and reference trials. A reference trial that follows a comparison trial requires switching the gate from closed to open in order to allow WM updating. Therefore, performance decrements when switching from a comparison to a reference trial are considered to reflect the behavioral cost associated with *opening* the gate to WM. Conversely, a comparison trial that follows a reference trial requires switching the gate from open to closed in order to allow WM maintenance. As such, worse performance when switching from a reference to a comparison trial is taken to indicate the behavioral cost associated with *closing* the gate to WM. In contrast, trial repetitions in terms of either reference or comparison trials are assumed to leave the state of the gate unaffected (i.e., the gate remains opened or closed, respectively; Rac-Lubashevsky & Kessler, 2016b).

By means of the reference-back paradigm, several recent studies have tried to elucidate the neural basis of the aforementioned WM subprocesses by investigating their neurophysiological correlates (Nir-Cohen et al., 2020; Rac-Lubashevsky & Kessler, 2018, 2019; Rempel et al., 2021). Overall, the results of these studies have provided evidence, broadly consistent with the PBWM model (Frank et al., 2001; Hazy et al., 2006), that updating and gating are dissociable processes characterized by their own EEG signature (Rac-Lubashevsky & Kessler, 2018, 2019; Rempel et al., 2021), and that gate opening, but not gate closing, depends on the activation of fronto-thalamic-striatal circuits (Nir-Cohen et al., 2020). Two more studies have investigated the dopaminergic basis of WM subprocesses (Jongkees, 2020; Rac-Lubashevsky et al., 2017) and provided indirect evidence for a more general involvement of striatal dopaminergic activity in WM updating and gate switching (Rac-Lubashevsky et al., 2017), and a more selective baseline-dependent dopaminergic modulation of gate opening (Jongkees, 2020) – both results in line with the critical role played by BG in regulating WM input gating, as assumed by the PBWM model (Frank et al., 2001; Hazy et al., 2006).

## The present study

Although the aforementioned literature has made significant progress on clarifying the neural basis of distinct WM processes assessed with the reference-back paradigm, to the best of our knowledge no studies have yet examined whether these processes are differentially sensitive to the kind of information that is to be encoded and maintained in WM. In the present study, we aim to extend our understanding of WM dynamics by examining whether and to what extent WM updating and gating can be affected by the relevance of the input to oneself.

A large number of studies has established that the social salience of a stimulus, namely whether the stimulus is relevant to, associated with, or owned by oneself (vs. others), can affect one’s cognitive functioning – a phenomenon that is referred to as a *self-bias or self-prioritization effect* (Cunningham & Turk, 2017; Sui et al., 2012; Sui & Gu, 2017; Sui & Humphreys, 2015a). Specifically, research has shown that, compared to *other*-associated stimuli, *self*-associated stimuli are perceived, processed (e.g., Keyes & Dlugokencka, 2014; Liu & Sui, 2016; Sui et al., 2012), and reach visual awareness faster (Macrae et al., 2017; but see Stein et al., 2016), are recalled better (e.g., Symons & Johnson, 1997; van den Bos et al., 2010), drive (exogenous and endogenous) attentional processing (e.g., Alexopoulos et al., 2012; Dalmaso et al., 2019; Golubickis & Macrae, 2021; Sui & Rotshtein, 2019; Truong & Todd, 2017; Zhao et al., 2015), modulate multisensory integration (e.g., Scheller & Sui, 2022), affect core aspects of cognitive control (e.g., Golubickis et al., 2021; Golubickis & Macrae, 2021; Svensson et al., 2022) and learning rate (e.g., Golubickis & Macrae, 2022; Lockwood et al., 2018), and bias perceptual decision making processing (e.g., Falbén et al., 2020; Golubickis et al., 2018). Most relevant for the purpose of the present study, two recent studies have shown a self-prioritization effect in WM (Yin et al., 2019, 2021). In a delayed match-to-sample WM task requiring participants to hold in mind different spatial locations, faster responses were observed when reacting to probes presented at locations where self-associated (as compared to other-associated) stimuli had previously appeared.

The delayed match-to-sample task is well suited to assess the temporal properties of WM in terms of how long participants can retain information in WM and/or how fast they can retrieve it. However, this paradigm does not allow for a decomposition of distinct WM processes as suggested by the PBWM model. That is, it remains unclear whether and to what extent input gating and updating processes are differentially modulated by self-relevance. In the present study we aim to fill this gap.

Mirroring the procedure adopted by Yin et al. (2019, 2021), we first trained participants to form specific associations between two neutral (arbitrary) stimuli and two social labels (oneself vs. a stranger, respectively) by performing a variant of the shape-label associative learning task developed by Sui et al. (2012). Immediately after, they performed the reference-back paradigm. In our version of the task, the shapes that were previously associated with the self or a stranger served as the stimuli that needed to be updated or maintained from trial to trial. Notably, the association between shape and label was irrelevant in the reference-back paradigm, where label did not play any role in determining the correct response per trial. This allowed us to examine if even a task-irrelevant association between stimuli and labels would influence the efficiency of WM processes acting on these stimuli.^1^

We based our hypotheses on the notion that the limited capacity of WM requires the cognitive system to scrupulously decide which information should be provided access to WM. This decision, i.e., gate opening, is a selective process that can be triggered by the presentation of salient stimuli, such as task-relevant information and reward cues (Kobayashi & Schultz, 2008; Ravizza & Conn, 2021; Schultz, 2013), which elicit phasic dopamine bursts in the basal ganglia (Frank et al., 2001; Hazy et al., 2007). Interestingly enough, self-bias effects are thought to be due to the fact that, once associated with the self, neutral stimuli acquire salience (Humphreys & Sui, 2015; see also Liu & Sui, 2016; Sun et al., 2016) and reward-related properties (Northoff & Hayes, 2011; Sui et al., 2012; Sui & Humphreys, 2015b; Yankouskaya, Bührle, et al., 2017a, 2017b). It is therefore reasonable to hypothesize that self-relevance may modulate selective gating of sensory information into WM.

**Fig. 1:**
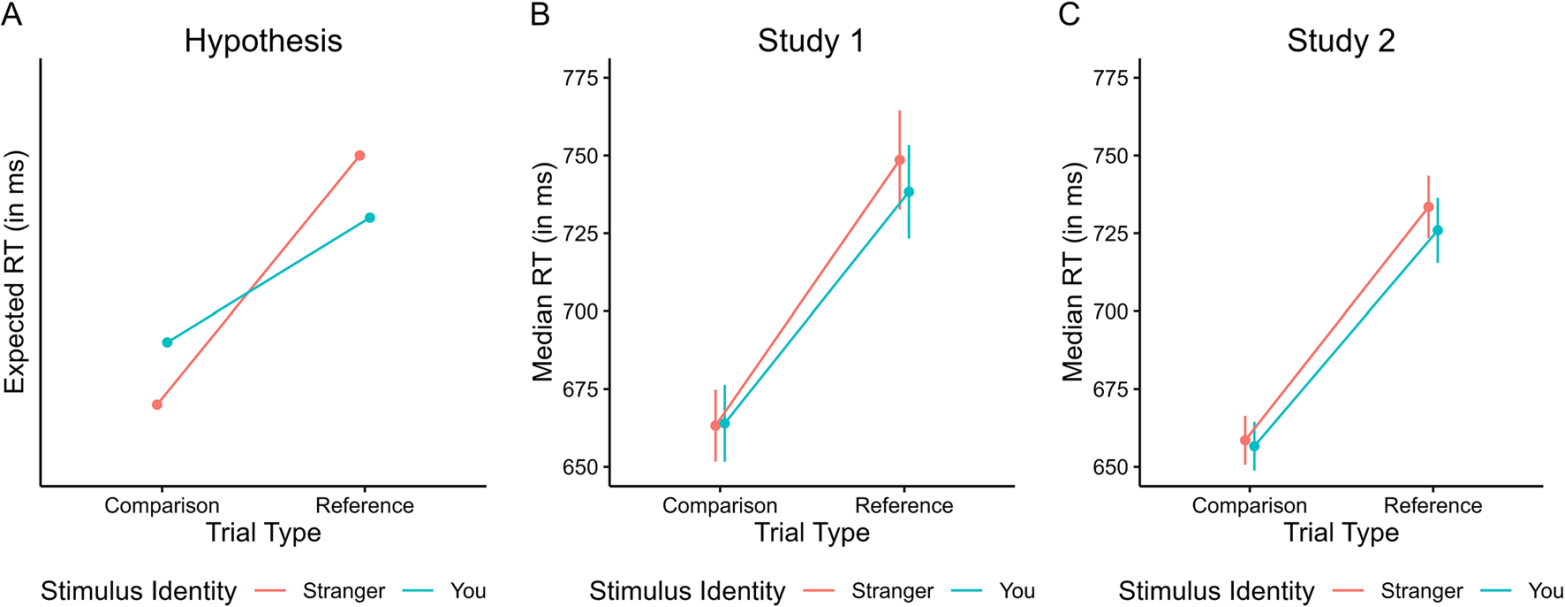
Visual representation of the primary hypothesis (**A**) and observed outcome in Study 1 (**B**) and Study 2 (**C**). Error bars indicate +/- 1 standard error of the mean

More specifically, we hypothesized that self-associated stimuli would facilitate WM gating due to their supposed inherently rewarding and salient properties. As such, our primary hypothesis was that performance would be facilitated when a self-associated feature is presented on a trial that requires WM gate opening and updating (i.e., on reference trials), as compared to when a stranger-associated feature is presented on such a trial. Following the same logic, we hypothesized that performance might actually be impaired when a self-associated feature is presented on a trial that requires WM gate closing and maintenance (i.e., on comparison trials), as compared to when a stranger-associated feature is presented on such a trial (see Fig. 1A). In addition, we explored whether the above-mentioned effects are modulated by (1) whether the currently maintained stimulus in WM is self versus stranger-associated, and (2) whether the previous trial required WM updating or maintenance.

To briefly anticipate findings regarding our primary hypothesis (Fig. 1B, C), we only observed a small non-significant difference in reaction time (RT) on reference trials involving a self-associated feature and no such difference on comparison trials. In our first of two studies we did observe a more complex pattern of results involving both the currently maintained stimulus in WM and trial-to-trial switching between WM updating and maintenance. Notably, this pattern of results was not replicated in a follow-up replication study (i.e., Study 2) involving a larger number of participants. As such, our studies do not provide evidence for a modulation of component processes of WM by social salience of stimuli. In the *General discussion* we speculate on reasons for this null-finding.

## Methods: Study 1

### Participants

To counterbalance the experimental design, a target number of 48 participants was chosen to double the sample size of previous work investigating self-prioritization effects in WM (Yin et al., 2019). Participants were recruited via the Leiden University research participation system and received two course credits for their participation in an online study on working memory. Individuals aged between 17 and 35 years, with no history of psychiatric disorders and drug use, normal or corrected-to-normal eyesight, and no color blindness were allowed to partake in the experiment. Overall, 57 young adults successfully completed the online study. However, 11 participants completed the experimental blocks of either task with less than 75% accuracy, which was the threshold we predefined in our ethics protocol (*n* = 4 and *n* = 8 for the associative learning task and reference back paradigm, respectively). In addition, one participant was identified as an outlier in terms of average RT in the reference-back paradigm. The data of all the above-mentioned participants were not further processed, resulting in a total sample size of 45 young adults (27 females, 17 males, one non-binary; mean age = 20.6 years, *SD* = 2.5, range = 17–28). This sample is sufficient to reliably detect a η_p_^2^ of 0.157 for the highest-order, four-way within-subject interaction effect with a power of 1 - β = .80 (α = .05; MorePower 6.0.4, Campbell & Thompson, 2012).

The study conformed to the ethics standards of the 1975 Declaration of Helsinki (as revised in 1983) and was approved by Leiden University’s local ethics committee (code 3203).

### Procedure

The experiment was carried out online by integrating the PsiTurk module (version 2.3.11; Gureckis et al., 2016) with custom-written JsPsych (version 6.1.0; de Leeuw, 2015) codes, mounted on an Ubuntu 20.04 (LTS) x64 server. Participants were asked to approach the experiment as if they were in a real lab setting and were instructed to perform the experiment in a quiet environment without any distractions (e.g., to put their mobile phone away or to activate silent mode), to close all applications that allow push messages and, if necessary, to wear glasses (or contact lenses). At the start of the experimental session, participants were asked to read the information letter, to indicate that they met the inclusion criteria, and to provide online informed consent. Participants were informed that they were free to withdraw from the study at any time. Before starting with the experimental tasks, participants indicated their age, gender, and whether their eyesight was (corrected to) normal. Thereafter the “credit card method” (Li et al., 2020; JsPsych resize-plugin) was used to estimate participants’ screen resolution and adjust stimulus size to be consistent across different screens. That is, participants were asked to hold a credit card (or another card of equal size) against an image of a credit card presented on-screen and to drag the corner of the credit card image until its size matched that of their real card. Hereafter, participants performed the associative learning task and then the reference-back paradigm.

The experimental session lasted approximately 60 min. At the end of the session, participants were debriefed and compensated for their participation.

### Materials

#### Shape-label associative learning task

**Fig. 2:**
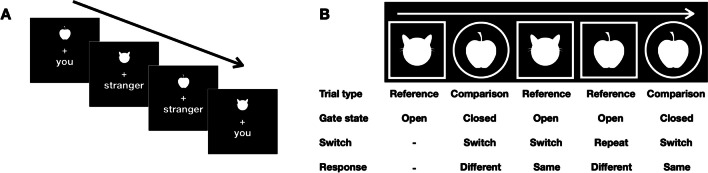
Schematic representation of a sequence of trials in the associative learning task (**A**) and reference-back task (**B**). (**A**) Participants were to indicate whether the presented shape-label pair matched the shape-label associations (e.g., apple-you vs. cat-stranger) that were to be memorized previously. (**B**) On each trial participants were instructed to indicate whether the currently displayed stimulus (i.e., apple or cat) was the same as or different from the stimulus most recently shown in a square frame (the cue shape – updating vs. maintenance mapping was counterbalanced between participants)

The task was adapted from Sui et al. (2012) and comprised a learning phase followed by a test phase. In the learning phase, participants were instructed to memorize specific associations between two arbitrary shapes (an apple vs. a cat, each 150 × 150 pixels) and two social labels (you vs. stranger, 69/99 × 69 pixels). The to-be-memorized shape-label pairs were presented in white on a black background for a duration of 60 s (“You are: ” and “A stranger is: ” presented sideways with the shapes), after which participants were to report the shape-label pairs by answering open-ended questions (e.g., “You are a(n): ”). On an incorrect response, the learning phase was repeated. Upon successful completion, the test phase followed to assess the presence of a self-prioritization effect (see Fig. 2A). This phase consisted of a practice block of 17 trials and four experimental blocks of 97 trials each, with the first trial of each block being a filler trial. Because the timings in the associative learning task are very rapid (see below), we required participants to complete the practice block with an accuracy higher than 70%. If this threshold was not met, the practice phase was repeated. For participants who did not reach this accuracy level on three consecutive tries, the experiment was terminated.

Each trial started with the presentation of a white fixation cross (31 × 31 pixels) for 495 ms, followed by a 195-ms on-screen presentation of one of the possible shape-label pairs (e.g., apple-self; both in white on a black background). For each pair, the shape was presented above the centrally presented fixation cross, while the label was presented below the cross. The distance between the center of the fixation cross and both the shapes and labels was 137 pixels. After the shape-label pair was removed from the screen, participants were to indicate, as fast and as accurately as possible, whether the presented pair matched or not the shape-label pair memorized during the practice phase, by pressing one of two response keys (the “V” or “B”, with “V” indicating a “match” response for 27 participants and a “non-match” response for 18 participants) on a QWERTY keyboard. Participants were allowed a maximum time of 1,095 ms to respond, which was succeeded by a 295-ms inter-trial interval. Within the practice block, this interval served as a window in which performance-contingent feedback was provided (green checkmark for correct responses, 120 × 120 pixels; a red crosshair for wrong responses, 120 × 120 pixels; and “too slow” for missed responses, 40-pixel red colored font). Within each of the blocks, each of the shapes (apple vs. cat) and labels (you vs. stranger) were presented equally often. The shape-label associations were counterbalanced across participants, and resulted in 21 participants associating themselves with a cat and a stranger with an apple, and the remaining (*n* = 24) undergoing the opposite mapping. The task lasted approximately 10 min.

#### Reference-back paradigm

To isolate WM processes and to investigate if these processes are affected by self-relevance, we used the reference-back task (see Rac-Lubashevsky & Kessler, 2016a, 2016b). Our version of the task involved the presentation of a sequence of the two shapes used in the shape-label associative-learning task (an apple and a cat), shown in white on a black background (see Fig. 2B). Stimuli were presented within white frames that varied in terms of shape (circle or square; all stimuli comprised 250 × 250 pixels). Participants were instructed to respond to the identity of the currently shown stimulus (e.g., the apple or cat) by indicating whether it was the same as or different from the most recent stimulus shown in, for example, a square frame. As such, stimuli that were presented in a circle frame required WM maintenance (i.e., the so-called comparison trials), as the most recently shown stimulus in a square frame remained unchanged. By contrast, stimuli that were presented in a square frame required WM updating (i.e., the so-called reference trials), as from that trial onwards the most recently-shown stimulus in a square frame was the currently-shown stimulus. For 27 participants, the square and circle frames signaled reference trials and comparison trials, respectively; for the other 18 participants, the mapping was reversed. For each of the blocks, the trial type (comparison vs. reference), trial-type switching (switch vs. repeat), and match-type (match vs. mismatch) levels were presented equally often.

Participants first performed one practice block of 33 trials to get acquainted with the task. Because accuracy tends to be very high in the reference-back paradigm (e.g., see Jongkees, 2020), we required participants to complete the practice with more than 80% accuracy before continuing to the experimental blocks, to ensure that participants were seriously engaged with the task. If participants did not reach this accuracy threshold after performing the practice block three times, the experiment was terminated. After the practice block, participants completed ten blocks of 65 trials each. The first trial of each block lasted 2,000 ms; it required no response and served as the first reference stimulus. The remaining trials within each block involved the presentation of a white fixation cross for 1,000 ms, followed by the simultaneous presentation of the target and the cue frame until a response was made. Participants indicated their matching decision by pressing the “P” button for “same” and the “Q” button for “different” on a QWERTY keyboard. The task lasted approximately 30 min.

### Statistical analyses

All analyses were performed using the statistical software R (R Core Team, 2019; Version 4.0.3). The alpha value for significance was set to .05, and the Benjamini-Hochberg correction was applied to *p* values to account for multiple comparisons (i.e., *p*_*corr*_ ; Benjamini & Hochberg, 1995).

For the associative learning task, both error rates (ERs) and median RTs were analyzed after removal of the first trial of each block, which served as a filler trial. For the RT analyses, error trials were also removed (11.66% of trials across all participants). Both ER and median RT were subjected to a repeated-measures ANOVA (*ezANOVA* from the R package *ez*) with Stimulus Label (*you* vs. *stranger*) and Matching Judgment (match vs. non-match) as within-subjects factors.

For the reference-back task, both ER and median RT were analyzed. The first trial of each block was excluded as it required no response. For the RT analyses, error (7.10% of all trials) and post-error trials were additionally removed (totaling 13.66% of trials). We first assessed whether basic reference-back effects could be replicated in our sample. To this end, both ER and median RT were subjected to a repeated-measures ANOVA. Trial Type (comparison vs. reference trials), Trial Type Switching (switch vs. repeat of WM trial type), and Match Type (match vs. mismatch) served as within-subject factors. Then, to assess whether the shape-label associations previously formed within the associative learning task affected reference-back performance, we conducted another repeated-measures ANOVA on ER and median RT^2^, which additionally included the following within-subject factors: Current WM Referent (*you*-associated vs. *stranger*-associated) and Current Target Stimulus (*you*-associated vs. *stranger*-associated). The factor Match Type was omitted in this case, as it became redundant with the Current WM Referent and Current Target Stimulus factors. For the RT analyses, planned paired *t*-tests were performed on the *updating*, *gate opening*, and *gate closing* costs in order to simplify higher-order interactions and more directly investigate the component processes of WM (see Table 1). Note that updating costs are calculated only for repeat trials, to avoid a confounding influence of gate switching in this contrast.

**Table 1:** Working memory (WM) components in the reference-back task as derived from the factors Trial Type (comparison vs. reference trials) and Trial Type Switching (switch vs. repeat of WM trial type). The signs (- vs. +) indicate how these orthogonal contrasts are calculated (e.g., *gate-opening* costs defined as the difference between reference-switch and reference-repeat trials, irrespective of the match/mismatch type)

	Comparison trials		Reference trials	
	Switch	Repeat	Switch	Repeat
Updating		-		+
Gate opening			+	-
Gate closing	+	-		

## Results: Study 1

### Shape-label associative learning task

**Fig. 3:**
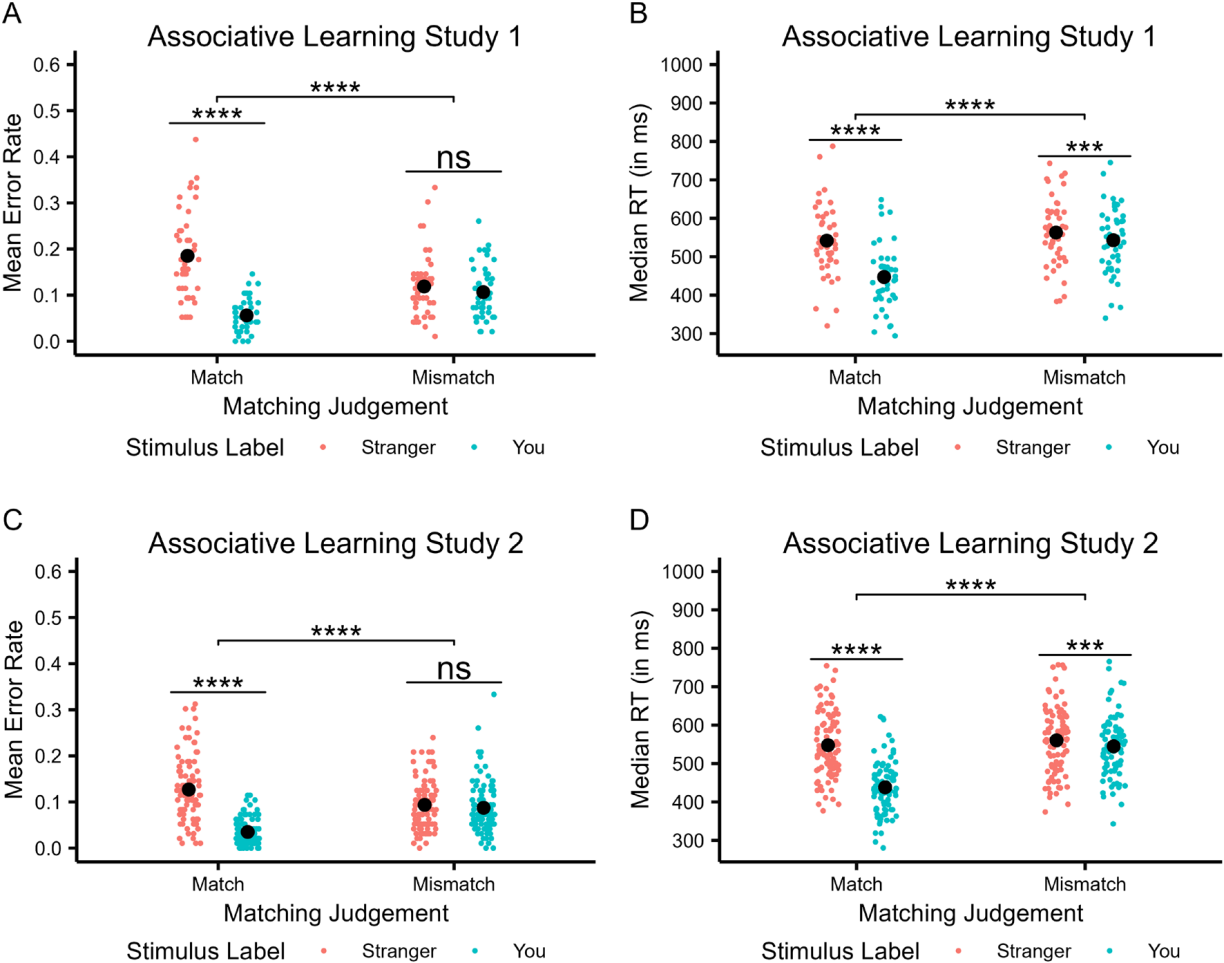
Associative learning task performance. Upper panels show mean error rates (ERs) (**A**) and median reaction times (RTs) (**B**) in Study 1, lower panels show mean ERs (**C**) and median RTs (**D**) in Study 2. Error bars representing +/- 1 standard error of the mean are too small to be visible

The ER analysis showed a main effect of Stimulus Label, *F*(1, 44) = 93.05, *p* < .001, η_p_^2^ = .68, with ER being smaller in response to *you* (*M* = 0.08, *SD* = 0.05) than to *stranger* trials (*M* = 0.15, *SD* = 0.09), while comparable ERs were observed for matched (*M* = 0.12, *SD* = 0.10) and non-matched pairs (*M* = 0.11, *SD* = 0.06), *F*(1, 44) = 1.08, *p* = .30, η_p_^2^ = .02. Moreover, these factors significantly interacted, *F*(1, 44) = 44.53, *p* < .001, η_p_^2^ = .50. For matched trials, ERs were smaller in response to *you* (*M* = 0.06, *SD* = 0.04) than to *stranger* trials (*M* = 0.19, *SD* = 0.10), |*t*|(44) = 10.03, *p*_*corr*_ < .001, *d* = 1.50, 95% CI [0.10, 0.16], whereas no such difference was observed for non-matched judgments, |*t*|(44) = 1.26, *p*_*corr*_ = .22, *d* = 0.19, 95% CI [-0.01, 0.03], where ERs in response to *you* (*M* = 0.11, *SD* = 0.06) and *stranger* trials (*M* = 0.12, *SD* = 0.07) were comparable (see Fig. 3A).

The RT analysis revealed significant main effects of Stimulus Label, *F*(1, 44) = 157.24, *p* < .001, η_p_^2^ = .78, and Matching Judgment, *F*(1, 44) = 136.28, *p* < .001, η_p_^2^ = .76, with RTs being faster when responding to *you* (*M* = 495, *SD* = 97) than to *stranger* trials (*M* = 552, *SD* = 92), and to matched (*M* = 494, *SD* = 100) than to non-matched pairs (*M* = 553, *SD* = 89). Furthermore, a significant interaction was observed, *F*(1, 44) = 46.16, *p* < .001, η_p_^2^ = .51. For matched trials, RTs were shorter in response to *you* (*M* = 447, *SD* = 82) than to *stranger* trials (*M* = 542, *SD* = 93), |*t*|(44) = 10.59, *p*_*corr*_ < .001, *d* = 1.58, CI = [77, 113], whereas a numerically smaller, yet significant difference was observed for the non-matched judgments, |*t*|(44) = 4.26, *p*_*corr*_ < .001, *d* = 0.64, CI = [11, 30], where RTs in response to *you* trials (*M* = 543, *SD* = 88) were shorter than RTs in response to *stranger* trials (*M* = 563, *SD* = 89). Overall, these results indicate that the manipulation was successful in inducing a robust self-prioritization effect (see Fig. 3B).

### Reference-back paradigm

#### Replication of basic effects – ERs

The analysis revealed significant main effects of Trial Type, *F*(1, 44) = 8.43, *p* = .006, η_p_^2^ = .16, and Match Type, *F*(1, 44) = 19.28, *p* < .001, η_p_^2^ = .31. Overall, ERs were lower on comparison (*M* = 0.07, *SD* = 0.06) than on reference trials (*M* = 0.08, *SD* = 0.07), and on match (*M* = 0.06, *SD* = 0.06) than on mismatch trials (*M* = 0.08, *SD* = 0.07). No such difference was observed for Trial Type Switching, *F*(1, 44) = 0.30, *p* = .59, η_p_^2^ < .01, indicating comparable ERs on repetition (*M* = 0.07, *SD* = 0.06) and switch trials (*M* = 0.07, *SD* = 0.07). Moreover, we observed a significant Trial Type × Trial Type Switching interaction, *F*(1, 44) = 7.84, *p* = .008, η_p_^2^ = .15, and Trial Type Switching × Match Type interaction, *F*(1, 44) = 16.84, *p* < .001, η_p_^2^ = .28. Overall, smaller ER switch costs (switch minus repeat) were observed on comparison (*M* = -0.01, *SD* = 0.04) than on reference trials (*M* = 0.01, *SD* = 0.04), while smaller mismatch costs (mismatch minus match) were observed on switch (*M* = 0.03, *SD* = 0.06) compared to repetition trials (*M* = 0.01, *SD* = 0.05). All other components were non-significant, *F*s(1, 44) < 1.33, *p*s > .25, η_p_^2^ < 0.03.

#### Replication of basic effects – RTs

The analysis revealed significant main effects of Trial Type, *F*(1, 44) = 84.58, *p* < .001, η_p_^2^ = .66, Trial Type Switching, *F*(1, 44) = 80.71, *p* < .001, η_p_^2^ = .65, and Match Type, *F*(1, 44) = 139.30, *p* < .001, η_p_^2^ = .76. Overall, RTs were slower on reference (*M* = 738, *SD* = 201) than on comparison trials (*M* = 660, *SD* = 154), on switch (*M* = 720, *SD* = 185) than on repeat trials (*M* = 678, *SD* = 179), and on mismatch (*M* = 789, *SD* = 192) than on match trials (*M* = 609, *SD* = 120). Moreover, we observed a significant Trial Type × Trial Type Switching interaction, *F*(1, 44) = 10.27, *p* = .003, η_p_^2^ = .19, indicating larger switch costs on comparison (*M* = 63, *SD* = 71) than on reference trials (*M* = 22, *SD* = 77). These effects were qualified by a Trial Type × Trial Type Switching × Match Type interaction, *F*(1, 44) = 17.41, *p* < .001, η_p_^2^ = .28. Subsequent post hoc *t*-tests were performed to compare *updating*, *gate-opening*, and *gate-closing* costs across match and mismatch trials.^3^

The analyses revealed larger *updating* costs on mismatch (*M* = 160, *SD* = 98) than on match trials (*M* = 37, *SD* = 76), |*t*|(44) = 7.38, *p*_*corr*_ < .001, *d* = 1.10, 95% CI [90, 157], and larger *gate-closing* costs on mismatch (*M* = 87, *SD* = 72) than on match trials (*M* = 39, *SD* = 62), |*t*|(44) = 3.84, *p*_*corr*_ < .001, *d* = 0.57, 95% CI [23, 73]), whereas the opposite pattern was observed for the *gate-opening* costs (i.e., larger costs on match, *M* = 41, *SD* = 58, than on mismatch trials, *M* = 3, *SD* = 88; *t*(44) = 2.54, *p*_*corr*_ = .015, *d* = 0.38, 95% CI [8, 69]). Overall, these observations replicate previous findings in the literature (Rac-Lubashevsky & Kessler, 2016b), with the notable exception that matching effects for the *gate-opening* component were reversed (i.e., gate-opening costs were larger on match trials than on mismatch trials). In the following section we explore if and how this reversal might be driven by the self-relevance manipulation.

#### Self-relevance and WM processing – ERs

When we added the self-relevance factors to the ANOVA of ER described above, the analysis did not show main effects of Current WM Referent, *F*(1, 44) = 1.00, *p* = .32, η_p_^2^ = .02, nor Current Target Stimulus, *F*(1, 44) = 0.86, *p* = .36, η_p_^2^ = .02, while these factors did interact, *F*(1, 44) = 19.26, *p* < .001, η_p_^2^ = .30. Overall, this indicated a matching-like effect where larger ERs were observed when the Current WM Referent and Current Target Stimulus were different (i.e., a mismatch; *M* = 0.08, *SD* = 0.07) as compared to when they were the same (i.e., a match; *M* = 0.06, *SD* = 0.06). Moreover, we observed a significant Trial Type Switching × Current WM Referent × Current Target Stimulus interaction, *F*(1, 44) = 18.92, *p* < .001, η_p_^2^ = .30, suggesting that the matching-like effect described above was only significant on switch trials (*p*s < .001), not on repetition trials (*p*s > .45). All other ANOVA components involving Current WM Referent or Current Target Stimulus were non-significant, *F*s(1, 44) < 2.10, *p*s > .15, η_p_^2^ < 0.05.

#### Self-relevance and WM processing – RTs

When we added the self-relevance factors to the ANOVA of RTs described above, the analysis showed no main effects of Current WM Referent, *F*(1, 44) = 2.29, *p* = .14, η_p_^2^ = .05, nor Current Target Stimulus, *F*(1, 44) = 1.41, *p* = .24, η_p_^2^ = .03. However, the ANOVA did yield a significant Trial Type × Trial Type Switching × Current WM Referent × Current Target Stimulus interaction, *F*(1, 44) = 17.04, *p* < .001, η_p_^2^ = .28^4^. To simplify the interaction and identify how WM performance costs were modulated by self-relevance, we once again computed the *updating*, *gate-opening* and *gate-closing* costs and submitted them to three separate repeated-measures ANOVAs with Current WM Referent and Current Target Stimulus serving as within-subjects factors (see Table 2 for descriptive statistics of these costs per condition). These follow-up ANOVAs revealed no significant main effect of Current WM Referent for the *updating* and *gate closing* components, *F*s(1, 44) < 1.16, *p*s > .28, η_p_^2^ < 0.03. However, Current WM Referent did modulate the *gate opening* component, *F*(1, 44) = 9.95, *p* = .003, η_p_^2^ = .18, indicating reduced costs when Current WM Referent was *you*-associated (*M* = 5, *SD* = 96) as compared to *stranger*-associated (*M* = 50, *SD* = 103). By contrast, no significant main effect of Current Target Stimulus was obtained for any of the components, *F*s(1, 44) < 2.70, *p*s > .10, η_p_^2^ < 0.06. Finally, for each of these components, a significant interaction between these two factors was observed, *F*s(1, 44) > 7.58, *p*s < .009, η_p_^2^ > .14.

**Table 2:** Mean updating, gate-opening, and gate-closing costs (*SD*) in the reference-back task in Study 1

Current WM Referent	Current Target Stimulus	Updating	Gate opening	Gate closing
You	You	33 (94)	34 (62)	36 (74)
Stranger	Stranger	40 (84)	61 (98)	40 (71)
You	Stranger	173 (110)	-24 (114)	81 (92)
Stranger	You	141 (118)	39 (107)	87 (113)

Paired *t*-tests revealed a typical matching effect indicating that *updating* costs and *gate-closing* costs were larger when Current WM Referent and Current Target Stimulus were different (i.e., a mismatch) as compared to when they were the same (i.e., a match), *updating*: *M* = 157, *SD* = 115 vs. *M* = 37, *SD* = 89; *gate closing*: *M* = 84, *SD* = 102 vs. *M* = 38, *SD* = 72). However, the *updating* and *gate-closing* costs did not differ significantly between *you*-associated matches and *stranger*-associated matches (*updating*: |*t*|(44) = 0.44, *p*_*corr*_ = .66, *d* = 0.07, CI = [-37, 24]; *gate closing*: |*t*|(44) = 0.36, *p*_*corr*_ = .77, *d* = 0.05, CI = [-29, 21]), nor did the costs differ significantly between the mismatches (*updating*: *t*(44) = 1.77, *p*_*corr*_ = .10, *d* = 0.26, CI = [-4, 68]; *gate closing*: |*t*|(44) = 0.30, *p*_*corr*_ = .77, *d* = 0.04, CI = [-48, 36]).

**Fig. 4:**
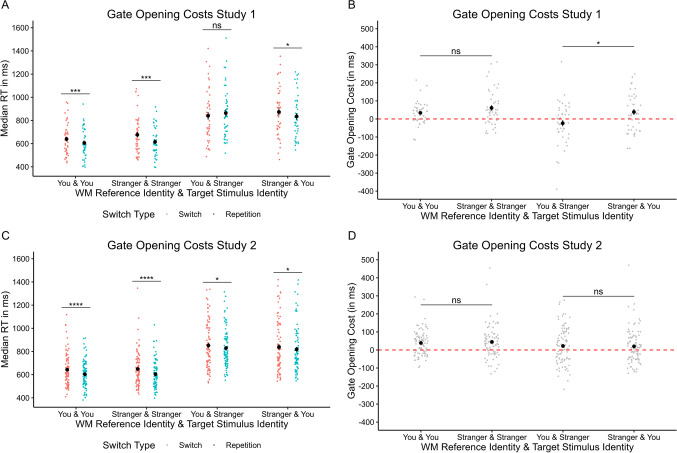
Decomposition of the *gate-opening* component (i.e., reference switch vs. reference repeat trials; panels **A** and **C**) and gate-opening costs (i.e., reference switch minus reference repeat trials; panels **B** and **D**) in the reference-back paradigm, for Study 1 (upper panels) and Study 2 (lower panels). Results of Study 1 reveal the absence of a repetition benefit on trials on which Current working memory (WM) Referent is *you*-associated and Current Target Stimulus is *stranger*-associated. This result was not replicated in Study 2, where a significant repetition benefit was observed across all conditions. Error bars indicate +/- 1 standard error of the mean

More importantly to the aim of the study, the analyses revealed a different pattern of results for *gate-opening* costs, which were still comparable between *you*-associated matches and *stranger*-associated matches, *t*(44) = 1.66, *p*_*corr*_ = .15, *d* = 0.25, CI = [-61, 6]. However, gate-opening costs were numerically reversed when Current WM Referent was *you*-associated and Current Target Stimulus was *stranger*-associated and significantly differed from when Current WM Referent was *stranger*-associated and Current Target Stimulus was *you*-associated, |*t*|(44) = 3.03, *p*_*corr*_ = .012, *d* = 0.45, 95% CI [-104, -21]). This pattern of results is illustrated in Fig. 4A, B, showing that the typical repetition benefit on consecutive reference trials is selectively eliminated when Current WM Referent was *you*-associated and Current Target Stimulus was *stranger*-associated, *t*(44) = 1.39, *p*_*corr*_ = .17, *d* = 0.21, 95% CI [-58, 10].

## Discussion: Study 1

The results of this study did not support our primary hypothesis, which entailed better performance when a *self*-associated (as compared to a *stranger*-associated) stimulus was presented on trials requiring WM gate opening and updating (i.e., on reference trials) as compared to trials requiring WM gate closing and maintenance (i.e., on comparison trials). While no modulation was observed in either the updating or the gate-closing components, the analyses suggested a potential impact of the self-relevance manipulation on WM gate-opening cost in RT. Specifically, the well-established repetition benefit on consecutive reference (i.e., updating) trials (Boag et al., 2021; Jongkees, 2020; Nir-Cohen et al., 2020; Rac-Lubashevsky & Kessler, 2016b) was eliminated when a self-associated stimulus was recently gated into WM and needed to be immediately replaced by a stranger-associated stimulus. Rather than indicating a non-specific facilitation of updating or maintaining self-associated stimuli, this pattern of results would suggest a selective impact of self-relevance on the efficiency of gate opening. To corroborate this conclusion, we conducted a direct replication of Study 1, employing a larger sample of participants (see below, Study 2).

If confirmed, these results would be consistent with and integrate literature on self-bias effects (e.g., Cunningham & Turk, 2017; Sui & Rothstein, 2019) and literature on component processes of WM (Rac-Lubashevsky & Kessler, 2016b; Rac-Lubashevsky & Frank, 2021). The observation that self-relevance modulates the selective gating of WM representations would be in line with the assumption that self-associated stimuli have higher intrinsic value than items associated with other people (e.g., Humphreys & Sui, 2015; Northoff & Hayes, 2011), and with the literature suggesting that WM gate opening can be triggered by the presentation of valuable stimuli such as task-relevant information and reward cues (Kobayashi & Schultz, 2008; Schultz, 2013; Ravizza & Conn, 2021). Such stimuli elicit bursts of phasic dopaminergic activity in BG, leading to gate opening (Frank et al., 2001; Hazy et al., 2007; Kobayashi & Schultz, 2008; Nir-Cohen et al., 2020; Ravizza & Conn, 2021; Schultz, 2013).

## Methods: Study 2

Methodological details were entirely identical to the first study. Participants were recruited via Academic Prolific and received $12 for their participation in an online study on WM. Overall, 110 participants successfully completed the online study. However, 17 participants completed the experimental blocks of either task with less than 75% accuracy, which was the threshold we predefined in our ethics protocol (*n* = 8 and *n* = 11 for the associative learning task and reference back paradigm, respectively). In addition, three participants were identified as an outlier in terms of average RT in the associative learning or reference-back paradigm. The data of all above-mentioned participants were not further processed, resulting in a total sample size of 90 young adults (44 females, 46 males, mean age = 25.8 years, SD = 4.5, range = 19–35). The shape-label associations were counterbalanced across participants, and resulted in 45 participants associating themselves with a cat and a stranger with an apple, and the remaining (*n* = 45) undergoing the opposite mapping.

For RT analyses in the associative learning task, the first trial of each block and error trials were removed (8.57% of all trials). For RT analyses in the reference-back task, the first trial of each block was also removed in addition to error (5.75%) and post-error trials (totalling 11.53% of trials).

## Results: Study 2

### Shape-label associative learning task

The ER analysis showed a main effect of Stimulus Label, *F*(1, 89) = 90.51, *p* < .001, η_p_^2^ = .50, and a main effect of Matching Judgment, *F*(1, 89) = 6.61, *p* = .012, η_p_^2^ = .07, with ER being smaller in response to *you* (*M* = 0.06, *SD* = 0.05) than to *stranger* trials (*M* = 0.11, *SD* = 0.07), and ER being smaller for matched (*M* = 0.08, *SD* = 0.07) than for non-matched pairs (*M* = 0.09, *SD* = 0.06). Moreover, these factors significantly interacted, *F*(1, 89) = 85.55, *p* < .001, η_p_^2^ = .49. For matched trials, ER were smaller in response to *you* (*M* = 0.04, *SD* = 0.03) than to *stranger* trials (*M* = 0.13, *SD* = 0.07), |*t*|(89) = 12.06, *p*_*corr*_ < .001, *d* = 1.27, 95% CI [0.08, 0.11], whereas no such difference was observed for non-matched judgments, |*t*|(89) = 1.03, *p*_*corr*_ = .31, *d* = 0.11, 95% CI [-0.01, 0.02], where ER in response to *you* (*M* = 0.09, *SD* = 0.06) and *stranger* trials (*M* = 0.10, *SD* = 0.05) were comparable (see Fig. 3C).

The RT analysis revealed significant main effects of Stimulus Label, *F*(1, 89) = 216.41, *p* < .001, η_p_^2^ = .71, and Matching Judgment, *F*(1, 89) = 397.96, *p* < .001, η_p_^2^ = .82, with RT being faster when responding to *you* (*M* = 492, *SD* = 90) than to *stranger* trials (*M* = 554, *SD* = 86), and to matched (*M* = 493, *SD* = 94) than to non-matched pairs (*M* = 553, *SD* = 82). Furthermore, a significant interaction was observed, *F*(1, 89) = 195.73, *p* < .001, η_p_^2^ = .69. For matched trials, RTs were shorter in response to *you* (*M* = 438, *SD* = 67) than to *stranger* trials (*M* = 548, *SD* = 85), |*t*|(89) = 17.46, *p*_*corr*_ < .001, *d* = 1.84, CI = [97, 122], whereas a numerically smaller, yet significant, difference was observed for the non-matched judgments, |*t*|(89) = 3.51, *p*_*corr*_ < .001, *d* = 0.37, CI = [3, 24], where RTs in response to *you* trials (*M* = 545, *SD* = 77) were shorter than RTs in response to *stranger* trials (*M* = 560, *SD* = 87). Overall, these results indicate that the manipulation was again successful in inducing a robust self-prioritization effect (see Fig. 3D).

### Reference-back paradigm

#### Replication of basic effects – ERs

The analysis revealed significant main effects of Trial Type, *F*(1, 89) = 21.11, *p* < .001, η_p_^2^ = .19, and Match Type, *F*(1, 89) = 34.50, *p* < .001, η_p_^2^ = .28, but not of Trial Type Switching, *F*(1, 89) = 2.30, *p* = .044, η_p_^2^ = .09. Overall, ER were lower on comparison (*M* = 0.05, *SD* = 0.05) than on reference trials (*M* = 0.06, *SD* = 0.06), and on match (*M* = 0.05, *SD* = 0.05) than on mismatch trials (*M* = 0.07, *SD* = 0.06), whereas ERs were comparable on repetition (*M* = 0.06, *SD* = 0.05) and switch trials (*M* = 0.06, *SD* = 0.06). Moreover, we observed a significant Trial Type × Trial Type Switching interaction, *F*(1, 89) = 23.44, *p* < .001, η_p_^2^ = .21, and Trial Type Switching × Match Type interaction, *F*(1, 89) = 23.16, *p* < .001, η_p_^2^ = .21. Overall, smaller ER switch costs (switch minus repeat) were observed on comparison (*M* = -0.02, *SD* = 0.05) than on reference trials (*M* = 0.02, *SD* = 0.05), while smaller mismatch costs (mismatch minus match) were observed on repetition (*M* = 0.004, *SD* = 0.05) compared to switch trials (*M* = 0.02, *SD* = 0.05). All other components were non-significant, *F*s(1, 89) < 0.42, *p*s > .52, η_p_^2^ < 0.01.

#### Replication of basic effects – RTs

The analysis revealed significant main effects of Trial Type, *F*(1, 89) = 158.59, *p* < .001, η_p_^2^ = .64, Trial Type Switching, *F*(1, 89) = 111.18, *p* < .001, η_p_^2^ = .555, and Match Type, *F*(1, 89) = 368.85, *p* < .001, η_p_^2^ = .81. Overall, RTs were slower on reference (*M* = 726, *SD* = 189) than on comparison trials (*M* = 655, *SD* = 142), on switch (*M* = 713, *SD* = 181) than on repeat trials (*M* = 668, *SD* = 157), and on mismatch (*M* = 778, *SD* = 178) than on match trials (*M* = 603, *SD* = 108). Moreover, we observed a significant Trial Type × Trial Type Switching interaction, *F*(1, 89) = 13.63, *p* < .001, η_p_^2^ = .13, indicating larger switch costs on comparison (*M* = 59, *SD* = 78) than on reference trials (*M* = 31, *SD* = 68). These effects were qualified by a Trial Type × Trial Type Switching × Match Type interaction, *F*(1, 89) = 52.28, *p* < .001, η_p_^2^ = .37. Subsequent post hoc *t*-tests were performed to compare *updating*, *gate-opening*, and *gate-closing* costs across match and mismatch trials.

The analyses revealed larger *updating* costs on mismatch (*M* = 145, *SD* = 93) than on match trials (*M* = 3255, *SD* = 66), |*t*|(89) = 12.9, *p*_*corr*_ < .001, *d* = 1.36, 95% CI [102, 139], and larger *gate-closing* costs on mismatch (*M* = 95, *SD* = 85) than on match trials (*M* = 23, *SD* = 50), |*t*|(89) = 7.84, *p*_*corr*_ < .001, *d* = 0.83, 95% CI [54, 90], whereas the opposite pattern was observed for the *gate-opening* costs (i.e., larger costs on match, *M* = 41, *SD* = 57, than on mismatch trials, (*M* = 21, *SD* = 77), *t*(89) = 2.31, *p*_*corr*_ = .023, *d* = 0.24, 95% CI [3, 37]). Overall, these findings are in line with the results of Study 1, in the sense that we replicated previous findings in the literature (Rac-Lubashevsky & Kessler, 2016b), with again the notable exception that matching effects for the *gate-opening* component were reversed (i.e., gate-opening costs were larger on match trials than mismatch trials). In the following section, we explore how this reversal relates to our self-relevance manipulation.

#### Self-relevance and WM processing – ERs

When we added the self-relevance factors to the ANOVA of ERs described above, the analysis did not show main effects of Current WM Referent, *F*(1, 89) = 2.69, *p* = .10, η_p_^2^ = .03, nor Current Target Stimulus, *F*(1, 89) = 0.44, *p* = .51, η_p_^2^ < .01, while these factors did interact, *F*(1, 89) = 32.22, *p* < .001, η_p_^2^ = .27. Overall, this indicated a matching-like effect where larger ERs were observed when the Current WM Referent and Current Target Stimulus were different (i.e., a mismatch; *M* = 0.06, *SD* = 0.07) as compared to when they were the same (i.e., a match; *M* = 0.05, *SD* = 0.06). Moreover, we observed a significant Trial Type Switching × Current WM Referent × Current Target Stimulus interaction, *F*(1, 89) = 24.22, *p* < .001, η_p_^2^ = .21, suggesting that the matching-like effect described above was only significant on switch trials (*p*s < .001), not on repetition trials (*p*s > .085). All other ANOVA interactions involving Current WM Referent or Current Target Stimulus were non-significant, *F*s(1, 89) < 3.31, *p*s > .07, η_p_^2^ < 0.04.

#### Self-relevance and WM processing – RTs

When we added the self-relevance factors to the ANOVA of RTs described above, the analysis showed no main effects of Current WM Referent, *F*(1, 89) = 1.75, *p* = .19, η_p_^2^ = .02, nor Current Target Stimulus, *F*(1, 89) = 2.12, *p* = .15, η_p_^2^ = .02. However, the ANOVA did yield a significant Trial Type × Trial Type Switching × Current WM Referent × Current Target Stimulus interaction, *F*(1, 89) =47.59, *p* < .001, η_p_^2^ = .35. To simplify the interaction and identify how WM performance costs were modulated by self-relevance, we once again computed the *updating*, *gate-opening*, and *gate-closing* costs (Table 3) and submitted them to three separate repeated-measures ANOVAs with Current WM Referent and Current Target Stimulus serving as within-subjects factors. These follow-up ANOVAs revealed no significant main effect of Current WM Referent for the *updating*, *gate-opening*, and *gate-closing* components, *F*s(1, 89) < 0.30, *p*s > .58, η_p_^2^ < 0.01, nor for Current Target Stimulus, *F*s(1, 89) < 0.61, *p*s > .43, η_p_^2^ < 0.01. Finally, for each of these components, a significant interaction between these two factors was observed, *F*s(1, 89) > 5.36, *p*s < .023, η_p_^2^ > .06.

**Table 3:** Mean updating, gate-opening, and gate-closing costs (*SD*) in the reference-back task in Study 2

Current WM Referent	Current Target Stimulus	Updating	Gate opening	Gate closing
You	You	23 (68)	39 (69)	17 (62)
Stranger	Stranger	32 (88)	45 (87)	25 (66)
You	Stranger	145 (110)	22 (103)	95 (99)
Stranger	You	142 (116)	20 (96)	94 (101)

Paired *t*-tests revealed a typical matching effect, indicating that *updating* costs and *gate closing* costs were larger when Current WM Referent and Current Target Stimulus were different (i.e., a mismatch) as compared to when they were the same (i.e., a match), *updating*: *M* = 143, *SD* = 113 vs. *M* = 27, *SD* = 79; *gate closing*: *M* = 94, *SD* = 99 vs. *M* = 21, *SD* = 64). However, the *updating* and *gate-closing* costs did not differ significantly between *you*-associated matches and *stranger*-associated matches (*updating*: |*t*|(89) = 0.98, *p*_*corr*_ = .40, *d* = 0.10, CI = -27, 9]; *gate closing*: |*t*|(89) = 0.92, *p*_*corr*_ = .43, *d* = 0.10, CI = [-26, 9]), nor did the costs differ significantly between the mismatches (*updating*: *t*(89) = 0.22, *p*_*corr*_ = .82, *d* = 0.02, CI = [-21, 26]; *gate closing*: |*t*|(89) = 0.84, *p*_*corr*_ = .93, *d* = 0.01, CI = -19, 20]).

More importantly to the aim of the study, the analyses again revealed a different pattern of results for *gate-opening* costs. Notably, the matching effect entirely disappeared, as none of the possible combinations of Current WM Referent and Current Target Stimulus differed significantly from each other, *t*s(89) ≤ 2.19, *ps*_*corr*_ > .19. Post hoc paired *t*-tests revealed that gate-opening costs were still significant for each combination of Current WM Referent and Current Target Stimulus, *t*s(89) > 2.00, *p*s_*corr*_ < .049, *d*s > 0.21. This pattern of results is illustrated in Fig. 4C, D.

## Discussion: Study 2

Study 2 replicates the absence of any influence of self-relevance on the updating and gate-closing components. However, it failed to replicate the pattern of results observed in gate-opening costs in Study 1. In this second study, comparable and statistically significant gate-opening costs were observed regardless of the self-relevance of the currently maintained stimulus in WM and the target stimulus. Considering that Study 2 was a direct replication attempt with double the sample size of Study 1 and therefore had higher statistical power, we suspect the gate-opening modulation observed in the first study occurred by chance. Therefore, the overall pattern of results leads us to conclude that self-relevance, as manipulated and incorporated in our experimental design, does not modulate distinct component processes of WM. However, we note that in our design the social saliency of the stimulus was entirely irrelevant to the WM task. As elaborated on in the next section, we speculate that different results might be obtained when social saliency is made to be task relevant in the WM paradigm.

## General discussion

Guided by recent observations that self-referential stimuli are prioritized in WM (Yin et al., 2019, 2021), the present study aimed to systematically investigate the potential of self-relevant information to modulate individual component processes of WM (i.e., maintenance vs. updating vs. gate opening/closing). To this end, participants first performed an associative learning task in which they associated two neutral shapes with two social labels (i.e., “you” vs. “stranger,” respectively). Immediately after, they performed the reference-back paradigm wherein the shapes that were previously associated with *you* or a *stranger* served as target stimuli. In brief, we hypothesized that stimuli associated with the self might be perceived as inherently more salient and/or rewarding (Humphreys & Sui, 2015; Northoff & Hayes, 2011; Sui et al., 2012), and therefore facilitate the updating/gating of these stimuli into WM or make it harder to prevent the updating of these stimuli on trials requiring WM maintenance.

However, across two studies we did not observe a modulation of either the updating or gate-closing components. Study 1 suggested a potential impact of self-relevance specifically on gate opening, as the facilitation of gate opening on consecutive trials disappeared when self-associated stimuli were recently gated into WM and then immediately needed to be replaced by stranger-associated stimuli. We tentatively speculated that this could suggest that gate opening became more effortful when you-associated stimuli were recently gated into WM. However, we did not replicate this pattern of results in a direct replication attempt with a larger sample size, suggesting this finding in Study 1 occurred by chance. Therefore, taken together, our results do not provide convincing evidence that self-relevance can affect distinct component processes of WM.

Our findings may seem inconsistent with earlier research on self-bias effects (e.g., Cunningham & Turk, 2017; Sui & Rothstein, 2019), particularly with those studies showing biased allocation of attention to self-associated stimuli during WM maintenance in a delayed match-to-sample task (Yin et al., 2019, 2021). These studies have suggested a tendency for self-referential stimuli to be prioritized in WM, even at the cost of overall performance. Furthermore, recent studies have shown that self-relevance can modulate various aspects of cognitive control (Dignath et al., 2022; Friehs et al., 2022; Golubickis & Macrae, 2021; Sui & Rothstein, 2019; Svensson et al., 2022). For example, recent work indicates enhanced conflict resolution in flanker-like tasks following the presentation of self-associated versus friend-associated cues (Svensson et al., 2022) and in response to self-associated and self-owned versus friend-associated and friend-owned objects (Golubickis & Macrae, 2021), with both studies using computational modeling to attribute these effects to a narrowing of visual attention. Similarly, another study has shown more efficient inhibitory control when responding to self-owned objects as compared to friend-owned objects (Golubickis et al., 2021; see also Friehs et al., 2022).

It is worth noting, however, that our experimental design differs from these previous studies in a subtle but potentially important way. Typically, studies investigating self-prioritization effects continuously reinforce associations between social labels and neutral stimuli by integrating these labels as task-relevant features or by requiring participants to explicitly recall these associations (e.g., Golubickis & Macrae, 2021; Golubickis et al., 2021; Svensson et al., 2022; Yin et al., 2019, 2021). For example, Yin et al. (2019, 2021) used a similar associative learning procedure to that in our study but using colors instead of shapes, and their design required participants to constantly remember these color-label associations in the subsequent delayed match-to-sample WM task. In that task, participants were instructed to memorize the location where a colored dot (previously associated with one of three social labels) appeared, and to compare it with the location of a subsequently presented probe. After each match trial, a label word was presented (Self, Friend, Stranger) and participants needed to judge whether the label matched the color of the dot presented at that location. Golubickis and Macrae (2021), instead, removed the need for an associative learning phase before a subsequent task by simply informing participants that the computer arbitrarily assigned them to one of two possible shapes and a stranger to the remaining one. These shapes served then as stimuli in a task displaying an array of three shapes (i.e., a central target and two flankers) and requiring participants to classify the social identity (self vs. stranger) of the target (for a similar procedure, see, e.g., Golubickis et al., 2021; Svensson et al., 2022). In contrast, in our design the label-stimulus associations were not necessary for performing the WM task nor were the associations reinstated in any way through reminders. In other words, the label-stimulus associations were completely irrelevant to the task at hand. This distinction in methodology might explain the absence of a significant impact on WM performance in our study. It potentially suggests that self-relevant stimuli, as incorporated in previous studies, influence performance only when they are directly relevant to the task or when they are actively maintained during task execution. This possibility is consistent with the results of recent work showing that self-prioritization is not obligatory, but emerges only when the task sets or the instructions make the previously established associations salient and available (Falbén et al., 2019; see also Macrae et al., 2017; Sui et al., 2012). Therefore, an interesting avenue to explore within future research would be whether a modulation of WM component processes can be observed when social associations are kept active throughout the task, for instance, by presenting continuous reminders of the label-stimulus associations in between blocks of the reference-back task, or by making the social associations more explicit by presenting possessive adjectives or pronouns alongside the target stimuli (e.g., my cat vs. your cat; cf. Dignath et al., 2022), or by making social associations task relevant by asking participants to recall the associated social labels when making the matching judgment in the reference-back task (cf. Yin et al., 2019, 2020).

## Limitations and future directions

Future work should try to address an important challenge in incorporating self-relevance into the reference-back task. In our studies, we mapped social identities (“you” vs. “stranger”) one-to-one on the two stimuli used in the reference-back task; this introduces a potential confound in trial-to-trial switching between identities and responses. For example, consecutive comparison trials where identity of the target stimulus alternates will necessarily involve a “same” response on one trial but a “different” response on the other trial, i.e., a response switch, because on consecutive comparison trials the stimulus in WM against which the matching decision is made does not change. This confound may have weakened our manipulation of self-relevance, reducing the likelihood of observing a significant effect. One way to de-confound our design is to assign multiple stimuli per identity, so that stimulus, response, and identity can vary independently from trial to trial. However, in systematically addressing this limitation, multiple sources of variability in results must be considered. First, to the best of our knowledge, shape-label associative learning tasks do not bind the same identity (e.g., “you”) to multiple, completely distinct stimuli, and thus we cannot be sure that self-prioritization effects might be even weaker when multiple stimuli are used. This binding of the self to multiple stimuli is perhaps more easily achieved in the well-established ownership paradigm (Cunningham et al., 2008), where an identity can be assigned ownership over multiple objects. Since self-owned objects are known to increase in subjective value (Humphreys & Sui, 2015; Morewedge & Giblin, 2015; Northoff & Hayes, 2011), this manipulation would fit nicely into our reasoning that associating an object with the self might interact with a value-driven WM gating system. Additionally, the reference-back literature has often only used two stimuli; it is unclear if and how performance on the reference-back task (e.g., the magnitude of gate opening and updating costs) change when more than two stimuli are used. All these considerations emphasize the importance of replicating the current study by integrating a systematic, incrementally expanding approach that can address these confounds.

Lastly, we based our hypotheses on the notion that self-associated stimuli are rewarding, i.e., they acquire salience and value (Humphreys & Sui, 2015; Northoff & Hayes, 2011; Sui et al., 2012), and that this would affect a BG-mediated gating mechanism. However, it should be noted that self-prioritization effects cannot be reduced entirely to reward: direct comparison of self-bias and monetary reward effects indicates similar yet separable neural correlates (e.g., Yankouskaya, Humphreys, et al., 2017a, 2017b) and self-bias effects differ in magnitude from reward (e.g., Yankouskaya, Bührle, et al., 2017a, 2017b; Yankouskaya et al., 2022). It has also been hypothesized that self-bias effects could be explained through attentional prioritization of positive emotions; however, careful comparison revealed distinct topological clusters of neural activation for self-bias versus positive emotion processing (Yankouskaya & Sui, 2021). Further highlighting a distinct effect of the self is the observation that individual differences in the effects of the self, reward, and emotion do not correlate (Yankouskaya et al., 2020, 2022). In light of these similarities and distinctions, it is interesting to note that Berridge and Robinson (2003) have proposed reward can be parsed into three dissociable components, namely, a learning, affective/hedonic, and motivational component. The learning component, also called reinforcement, refers to people’s tendency to repeat a behavior that was rewarded through associative conditioning. The affective/hedonic component concerns the positive feelings (“pleasure”) experienced when receiving a reward. The motivational component refers to people’s willingness to exert cognitive efforts (“wanting” and “incentives”) when reward is promised for good performance. We speculate that perhaps the distinct effects of the self, emotions, and monetary rewards can be reconciled through linking these effects to the learning, affective/hedonic, and motivational components of reward, respectively. To further investigate the unity and diversity of these different effects, future work may assess item-specific effects of emotions and monetary reward on component processes of WM and compare them with the self-bias effects (or lack thereof).

## Conclusion

Across two studies we did not find evidence for the hypothesis that the self-relevance of a stimulus can modulate distinct component processes of WM, as isolated in the reference-back paradigm. However, it is premature to entirely dismiss this possibility. More research is warranted to address the limitations of the present study and gain insights into the specific conditions that might allow the social saliency of a stimulus to affect cognitive performance. Integrating behavioral, computational, and neuroimaging methodologies in such a research line would be highly advisable (cf. Trutti et al., 2021).

## Footnotes

1 The project was originally more ambitious and involved a much more complex experimental design, which was later simplified after we carried out an experiment in which we failed to observe a self-relevance modulation of WM. Specifically, our initial aim included assessing if self-relevance impacted WM differently depending on whether self-relevance is bound to task-relevant versus task-irrelevant stimulus features of either the target stimulus or the cue frame in the reference-back task. This led us to implement a task design different from the one described in this article, wherein both the target stimulus and the cue frame varied from trial to trial in terms of two dimensions, one task-relevant and one task-irrelevant. However, after running a pilot and one study, we realized some suboptimal design choices had been made. As a result, we decided to simplify our investigation to the aim and design as reported here. We did not deem it appropriate to describe this earlier experiment in this work because some of the choices we made in experimental design are understandable only in the context of the more complex aim we had in mind and follow-up studies that we did not carry out. However, for the sake of transparency, we decided to acknowledge the existence of this former study and to provide a full description of the method and results of this study and its pilot, which can be found at https://osf.io/jygfn/.

2 The reported effects of interest replicate when analyzing trimmed mean RT; for each participant, data were trimmed by removing outlier trials that deviated more than 2.5 SD of a participant’s mean of the RT distribution (grouped by the factors Trial Type, Trial Type Switching, Current WM Referent, and Current Target Stimulus).

3 A fourth component that is typically extracted within the reference-back task is the so-called *substitution* cost, which reflects the cost of updating WM content with new information (on reference mismatch trials) rather than with information that is already maintained in WM (on reference match trials). The reported effect of Match Type on *updating* costs captures this *substitution* cost (here on average 160 − 37 = 123 ms).

4 To test whether the shape-label associations (*you*-apple vs. *you*-cat) might have differentially contributed to the reported effects, we conducted separate repeated-measures ANOVAs for both possible label-shape mappings. Crucially, for both mappings, the analyses showed a replication of the reported four-way interaction effect, both for median RTs, *F*s(1, 44) > 6.82, *p*s < .016, η_p_^2^ > 0.23, and for trimmed mean RTs, *F*s(1, 44) > 6.20, *p*s < .02, η_p_^2^ > 0.21.
